# Supplementary material for: The genome sequence of a mirid bug, Stenodema calcarata (Fallen, 1807)
Source: Wellcome Open Res. 2025 Feb 28;10:115. [Version 1] doi: 10.12688/wellcomeopenres.23763.1 (PMC11950723; doi:10.12688/wellcomeopenres.23763.1)
Supplement: Peer review history

# Reviewer report: Reviewer response for version 1

**DOI:** 10.21956/wellcomeopenres.26211.r120715

**Published:** 2025-03-27

**Authors:** Qianquan Chen

**Affiliations:** 1 Guizhou Normal University, Guiyang, China

*Stenodema calcarata *is a large grass bugs, which is widely distributed in the UK, however, the biology of *Stenodema* remain unknown. A chromosome-level genome sequence was decoded by using HiFi and Hi-C sequencing. Methods are presented clearly in the main text. The genome sequence can contribute to the research of *S. calcarata*.

Are sufficient details of methods and materials provided to allow replication by others?

Yes

Is the rationale for creating the dataset(s) clearly described?

Yes

Are the datasets clearly presented in a useable and accessible format?

Yes

Are the protocols appropriate and is the work technically sound?

Yes

Reviewer Expertise:

Eco-genomics

I confirm that I have read this submission and believe that I have an appropriate level of expertise to confirm that it is of an acceptable scientific standard.

# Reviewer report: Reviewer response for version 1

**DOI:** 10.21956/wellcomeopenres.26211.r120417

**Published:** 2025-03-24

**Authors:** Michelle Guerrero

**Affiliations:** 1 University of the Philippines, Los Baños, Philippines

Summary of the Article

 The article presents the genome assembly of *Stenodema calcarata*, a species of mirid bug in the Hemiptera order. The genome sequence spans 575.35 Mb, with 98.42% of the assembly scaffolded into 18 chromosomal pseudomolecules. Additionally, the mitochondrial genome has been assembled at 19.31 kb. The study details sequencing methodologies, assembly curation, and assessment of genome quality metrics.

 The study utilized Pacific Biosciences HiFi sequencing and Hi-C sequencing, generating a high-quality, chromosome-level assembly. Various quality control measures, including BUSCO completeness analysis, k-mer completeness, and Hi-C mapping, indicate a highly contiguous and accurate genome sequence. The study also provides extensive methodological details, including DNA extraction, library preparation, and assembly curation.

 Evaluation Criteria

 1. Is the rationale for creating the dataset(s) clearly described?

**Yes**

 The article provides a clear rationale for sequencing the genome of *Stenodema calcarata*, emphasizing its ecological importance, taxonomy, and specialization in grass-feeding. The study also highlights the potential for genomic insights into species delimitation and ecological adaptation. The introduction and background sections provide sufficient context for why this genome was selected for sequencing.

 2. Are the protocols appropriate and is the work technically sound?

**Yes**

 The methods employed for genome sequencing, assembly, and curation follow established and widely accepted protocols. The use of PacBio HiFi long reads ensures high accuracy, and Hi-C sequencing facilitates the generation of a chromosome-level assembly. Quality control metrics, including BUSCO completeness, k-mer analysis, and scaffold validation, support the robustness of the assembly. The manuscript demonstrates a high level of technical rigor in ensuring data accuracy and completeness.

 3. Are sufficient details of methods and materials provided to allow replication by others?

**Yes**

 The article provides an extensive methodological description, including sample collection, DNA extraction, sequencing, library preparation, assembly curation, and validation. References to standard operating procedures and publicly available protocols further enhance the reproducibility of the study. The detailed account of software tools and quality assessment measures ensures that other researchers can replicate or build upon this work.

 4. Are the datasets clearly presented in a usable and accessible format?

**Partly**

 The genome sequence and associated datasets are well-documented, with references to interactive visualizations and data repositories such as GenBank. However, the accessibility and usability could be improved by providing clearer instructions on how to access and download the datasets. Additionally, including a direct DOI or dataset link within the main text would enhance ease of use.

 Recommendations for Improvement

- **Clarify dataset access:** Ensure that direct links to all datasets are explicitly provided in the manuscript for easier access.
- **Enhance dataset usability:** Consider providing a supplementary table summarizing the dataset components, their repository locations, and how they can be accessed.
- **Minor clarification in curation steps:** While the assembly curation process is well-documented, it may benefit from a more structured summary of key changes and corrections made.

Are sufficient details of methods and materials provided to allow replication by others?

Yes

Is the rationale for creating the dataset(s) clearly described?

Yes

Are the datasets clearly presented in a useable and accessible format?

Partly

Are the protocols appropriate and is the work technically sound?

Yes

Reviewer Expertise:

Molecular Entomology, Metagenomincs

I confirm that I have read this submission and believe that I have an appropriate level of expertise to confirm that it is of an acceptable scientific standard.
